# Evaluation of artificial intelligent breast ultrasound on lesion detection and characterization compared with hand-held ultrasound in asymptomatic women

**DOI:** 10.3389/fonc.2023.1207260

**Published:** 2023-06-15

**Authors:** Bin Xu, Weidong Luo, Xin Chen, Yiping Jia, Mengyuan Wang, Lulu Tian, Yi Liu, Bowen Lei, Jiayuan Li

**Affiliations:** ^1^ Department of Epidemiology and Health Statistics, West China School of Public Health, Sichuan University, Chengdu, China; ^2^ West China Fourth Hospital, Sichuan University, Chengdu, China

**Keywords:** breast, hand-held breast ultrasound, automated breast ultrasound, screening, breast lesion

## Abstract

**Introduction:**

To compare the accuracy of Artificial Intelligent Breast Ultrasound (AIBUS) with hand-held breast ultrasound (HHUS) in asymptomatic women and to offer recommendations for screening in regions with limited medical resources.

**Methods:**

852 participants who underwent both HHUS and AIBUS were enrolled between December 2020 and June 2021. Two radiologists, who were unaware of the HHUS results, reviewed the AIBUS data and scored the image quality on a separate workstation. Breast imaging reporting and data system (BI-RADS) final recall assessment, breast density category, quantified lesion features, and examination time were evaluated for both devices. The statistical analysis included McNemar’s test, paired t-test, and Wilcoxon test. The kappa coefficient and consistency rate were calculated in different subgroups.

**Results:**

Subjective satisfaction with AIBUS image quality reached 70%. Moderate agreements were found between AIBUS with good quality images and HHUS for the BI-RADS final recall assessment (*κ* = 0.47, consistency rate = 73.9%) and breast density category (*κ* = 0.50, consistency rate = 74.8%). The lesions measured by AIBUS were statistically smaller and deeper than those measured by HHUS (*P* < 0.001), though they were not significant in clinical diagnosis (all < 3 mm). The total time required for the AIBUS examination and image interpretation was 1.03 (95% *CI* (0.57, 1.50)) minutes shorter than that of HHUS per case.

**Conclusion:**

Moderate agreement was obtained for the description of the BI-RADS final recall assessment and breast density category. With image quality comparable to that of HHUS, AIBUS was superior for the efficiency of primary screening.

## 1 Introduction

Hand-held ultrasound (HHUS), which is widely available and radiation-free, has gained acceptance as an important imaging modality to detect and characterize breast lesions (1, 2). Previous comparative studies have shown that breast ultrasound may outperform mammography in Asian women, who have dense breasts and are diagnosed with breast cancer at a younger average age than Western women (3–5). Therefore, ultrasound is an appropriate approach to address Chinese enormous need for primary breast cancer screening. Currently, China guideline recommends that the general female population should undergo breast ultrasound examination every 1-2 years between the ages of 45 and 70 (6). However, several limitations of the conventional ultrasound make it difficult to take full advantage of its use for large collectives. One of the main challenges HHUS encounters is the excessive amount of time spent by radiologists, particularly when the demand is high (7). Another issue that must be considered is the lack of standardization and reproducibility that would result from printing only subjectively selected screenshots during the examination (8). In addition, HHUS is strongly reliant on the skill and experience of the operators (9), who are in short supply in primary healthcare facilities (10).

To overcome these drawbacks, the concept of automated breast ultrasound as an alternative to HHUS was introduced in the 1970s and has since been developed as artificial intelligent breast ultrasound (AIBUS) (11). AIBUS consists of an automated scanner arm, one-touch buttons, and a matched bed to improve workflow through constant and complete routines to standardize and accelerate image acquisition. This ultimately allows it to deliver reproducible and standardized breast ultrasound images with less operator dependence. What’s more, the integrated storage of breast ultrasound video data enables additional examiners to perform comfortable and time-efficient second readings at any time. Decoupling of image acquisition and reading improves the possibilities of implementing breast ultrasonography in screening and follow-up evaluations. Medical assistants can simply carry out the full procedure, freeing the specialized radiologist to focus on the interpretation and diagnosis. Additionally, it is feasible to train health personnel of varying experience levels to acquire AIBUS images, which will cost far less than recruiting qualified radiologists.

Since these new devices offered a technically promising method for ancillary diagnosis of breast disease, it is necessary to ensure reliability in clinical practice. However, the diagnostic values of this new technique, such as lesion detection and breast description, have yet to be fully discussed in previous reports. It is critical to report accurate lesion recognition and characterization, including location, size, and feature description in the whole-breast scan. The anticipated malignancy probability evaluation in HHUS using the breast imaging reporting and data system (BI-RADS) lexicon of the American College of Radiology (ACR) has been demonstrated to be excellent (12). Management according to the BI-RADS final recall assessment is important, hence the clinical application of AIBUS to classify screening populations needs to be assessed beforehand.

In this proof-of-concept study, we assessed the technical feasibility of performing AIBUS in asymptomatic volunteers. This study aimed to (1) evaluate the overall image quality of AIBUS; (2) assess the reliability of AIBUS examinations in classifying screening women by the BI-RADS category results; and (3) compare the lesion detection and characterization between AIBUS with HHUS.

## 2 Method

### 2.1 General design and participants

**Figure 1:**
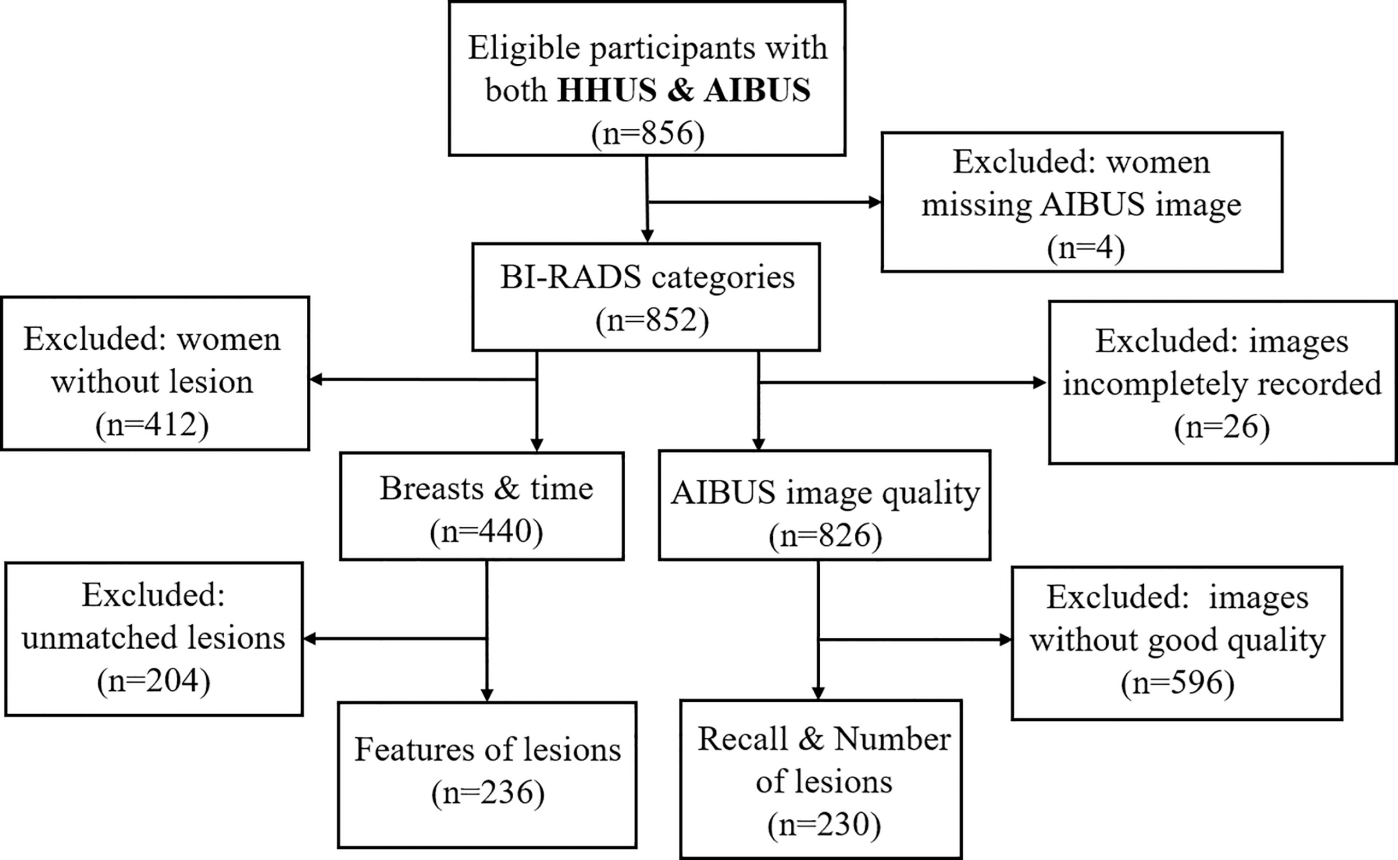
Study diagram presenting study sample selection. BI-RADS, breast imaging reporting, and data system; HHUS, handheld ultrasound; AIBUS, Artificial Intelligent Breast Ultrasound. Breast and time: Participants with lesions for whom breast features and time of examination were recorded. Unmatched lesions: Participants with lesions detected in HHUS and AIBUS that could not be matched to the same lesion by the radiologists after unblinding. Recalls and number of lesions: Participants who were recalled for further examination and the number of lesions was recorded. Features of lesions: Participants who had lesion characteristics recorded.

A unicentric cross-sectional study was carried out from December 2020 to June 2021 at the Ultrasound Department of West China Fourth Hospital in Chengdu, China. Asymptomatic adult females who voluntarily underwent both HHUS and AIBUS were included. Typically, the standard screening routine produced for volunteers included a conventional breast ultrasound and any necessary subsequent examinations. On the same day as the HHUS exam, volunteers also had an AIBUS exam. Women who were breastfeeding or had a history of breast implants or mastectomies were excluded. Recall was recommended for further examination if the final BI-RADS assessment up to 3 or 4 under any one of the two exams A final sample of 852 participants (age range, 20-75 years; mean, 40 years) was included. 440 of them (age range, 20-70 years; mean, 40 years) were detected with breast lesions (**Figure 1**).

### 2.2 Breast ultrasound

**Handheld device**—Breast ultrasound with the handheld device (Mindray Resona7), which was equipped with a 50-mm linear-array transducer with a bandwidth of 5-14 MHz, was randomly performed by one of two radiologists with 8 and 11 years of experience in breast imaging. The scanning technique for bilateral whole-breast ultrasound was standardized as follows: in the supine oblique position, the woman lay on the examination bed with her arms raised above her head. The right breast was scanned in a transverse and sagittal orientation, with a radial scan centered on the nipple, and then, the left breast was examined in the same way.

**Figure 2:**
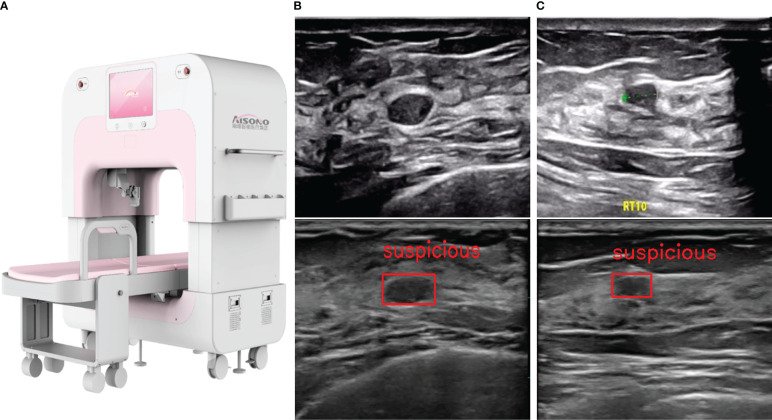
AIBUS ultrasound machine and images. **(A)** The machine appearance of AIBUS **(B)** HHUS (top) and AIBUS (bottom) image of example 1: a 46-year-old woman with breast mass that was interpreted as BI-RADS 3 **(C)** HHUS (top) and AIBUS (bottom) image of example 2: a 57-year-old woman with breast mass that was interpreted as BI-RADS 3.

**Automated system**—AIBUS (AISONO, AIBUS 100 pro) was conducted by a medical assistant, who completed a 7-day trial period session. The device was equipped with a flexible robot arm, vision system, control system, and touch display system (**Figure 2A**). The ultrasonic scanning arm with three orthogonal translational axes and two orthogonal rotating axes was used for large-range automatic simultaneous mammary ultrasonic scanning. The scanning technique was standardized as follows: participants were in a supine position with arms above the head, wearing a disposable membrane vest to secure the breasts and guarantee adequate contact with the skin. After the examiner chose the intensity of the scan depending on the size of the breast, the high-frequency transducer applied a modest compression to stabilize the breast. The ultrasonic probe scans along the route predetermined by the visual system, sliding 6 times in the coronal position on each side. The transducer with a 7.5-MHz center frequency captures a series of 720×440 high-resolution images up to 38 mm (width) ×59 mm (depth). Once the acquisition is complete, the entire set of scans was automatically forwarded to a separate workstation in the form of a video, with a frame rate of 18 frames per second and a size of about 4.5M (**Figures 2B, C**). The YOLOV1-V4 target detection technique is used to pinpoint the precise location of lesions. The lesion region of interest was identified in an image using 3D-CNN, and it was subsequently classified and shown using the visualization interface (13, 14). It provided comprehensive data processing and picture analysis facilities, while also being reviewed in a multi-planar reconstruction display.

### 2.3 Features recorded

Two radiologists independently interpreted all AIBUS images at the workstation in random order a month (washout period) after evaluating all HHUS images. Before this, they attended a 2-hour tutorial to learn how to use the workstation, and they reviewed and discussed breast ultrasound examinations during the 7-day trial period. The clinical details and conclusions shown on the corresponding HHUS images were kept blinding to them.

Each reader was required to assign lesions to BI-RADS categories: 0, incomplete (excluded from this study); 1, negative; 2, benign; 3, probably benign; 4A, low suspicion; 4B, intermediate suspicion; or 4C, moderate suspicion (not found in this study). BI-RADS 3 was the threshold for primary screening recall, whereby BI-RADS 3 and 4 denote additional diagnostic workup and BI-RADS 1 and 2 denote no additional diagnostic workup. Radiologists subjectively rated their satisfaction with AIBUS images on dimensions such as clarity, integrity, and effective image proportion. Each dimension was scored from 1 to 5, with 5 being the best, and a total of 12 points were of good quality. For each participant with a breast lesion detected on either HHUS or AIBUS, breast density category (dense or non-dense) and breast thickness were measured with an axial scan in the outer upper quadrant area and were recorded in millimeters. For each lesion detected on both HHUS and AIBUS, the size (long diameter and short diameter) and depth from the skin were measured in millimeters and centimeters, respectively. After unblinding, the two radiologists matched the same lesion found by both two devices for comparative analysis. The radiologist in charge of the AIBUS interpretation worked out agreements through discussion where there was a dispute. The acquisition and reading time of HHUS was calculated using an available stopwatch, starting when the participant was ready to be examined and ending with image interpretation. The execution time of the AIBUS machine was fixed at 3.5 minutes, and the same stopwatch was serviced to time the reading process from opening the study at the workstation until its completion.

### 2.4 Data review and statistical analysis

This explorative study was based on descriptive statistical methods. Mean with standard deviation and median with quartiles were given for data with normal and non-normal distribution, respectively. We contrasted the performance of AIBUS with the results of the conventional HHUS (gold standard). Paired sample t-test was applied to continuous variables, such as breast thickness, lesion size, lesion depth from the skin, and the elapsed time. Wilcoxon test was used to compare the paired BI-RADS categories. McNemar’s test was employed to assess breast density category and recall rate. To measure agreement, Kappa statistics, and consistency rates were calculated and estimated in different subgroups according to age and body mass index (BMI). To obtain the confidence intervals, we used 1000 bootstrap samples with replacement. A kappa value of > 0.60 denoted substantial agreement; 0.41-0.60, moderate agreement; 0.21-0.40, fair agreement; and 0.20 or less, slight agreement. Statistical significance was assumed as *P* < 0.05 for the two-tailed test.

### 2.5 Ethics approval statement

The current study was approved by the Institutional Review Board (IRB approval No. KS2020230). All participants provided signed informed consent.

## 3 Results

A total of 852 participants (age range, 20-75 years; mean, 40 years) received both conventional HHUS and AIBUS. According to the BI-RADS categorization, 32.0% (*n* = 273) of our participants were assigned as BI-RADS 1 by HHUS, 16.2% (*n* = 138) had lesions as BI-RADS 2, 38.6% (*n* = 329) as BI-RADS 3 and 13.1% (*n* = 112) as BI-RADS 4 (**Table 1**).

**Table 1:** Cross-tabulation of BI-RADS in detail by HHUS and AIBUS.

HHUS BI-RADS	AIBUS BI-RADS					Total
	1	2	3	4a	4b	
1	166	31	61	13	2	273
2	66	31	30	8	3	138
3	90	25	153	54	7	329
4a	20	7	28	47	6	108
4b	1	0	0	2	1	4
Total	343	94	272	124	19	852

AIBUS, Artificial Intelligent Breast Ultrasound; HHUS, hand-held ultrasound; BI-RADS, breast imaging reporting and data system.

### 3.1 The overall image quality of AIBUS

Technically speaking, the AIBUS device was reliable and all attempted scans were completed. 826 AIBUS images with complete quality evaluation were included. With an overall satisfaction rate of 70%, the radiologists’ subjective satisfaction with AIBUS image clarity, integrity, and the proportion of effective images achieved 73%, 68%, and 69% respectively (**Table S1**). 27.85% (230/826) of the images were rated as good quality.

### 3.2 BI-RADS category results for AIBUS

Wilcoxon rank-sum test was performed on the BI-RADS category of the 852 participants who underwent both examinations (**Table 1**), and no significant difference was found (*z* = -1.503, *P* = 0.133). The intra-group correlation coefficient between the two BI-RADS groups was 0.577 (*P* < 0.001), indicating a moderate correlation.

**Figure 3:**
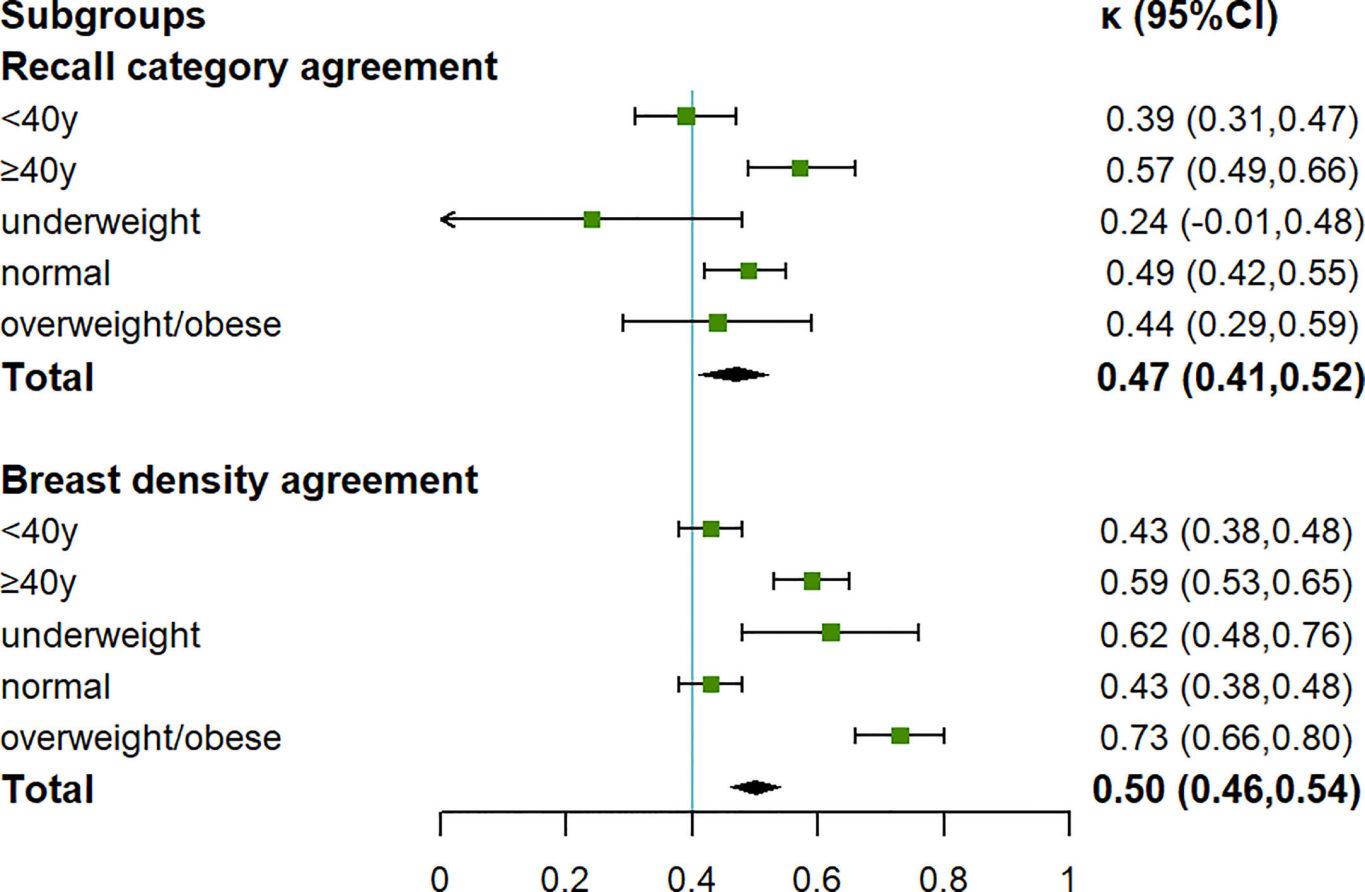
Agreement in final recall assessment and breast density category by subgroups.

230 pairs of ultrasound results with good image quality were included and analyzed in subgroups for the consistency of the further recall assessment (**Table 2**). Only in women under the age of 40, McNemar’s test showed different diversions between the two examinations (*P* = 0.04). The overall kappa value reached a moderate agreement (0.47, 95% *CI* (0.41, 0.52)), and among women over 40 it was the highest (0.57, 95% *CI* (0.49, 0.66)) (**Figure 3**). The overall consistency rate was 73.91%, with the highest consistency rate at 79.59% in women over 40 years old (**Table 2**). The lesion number of those 230 participants was paired for comparison (**Table S2**). The average number of lesions detected by HHUS and AIBUS was 0.84 (193/230) and 0.71 (164/230) respectively. 44.3% (102/230) patients were found to have breast lesions by HHUS and 37.8% (87/230) by AIBUS. The two devices detected a comparable overall number of lesions, according to the Wilcoxon test (*P* = 0.311).

**Table 2:** Comparison of final recall assessment between HHUS and AIBUS.

Subgroups	HHUS	AIBUS		Subtotal	*P*	Consistency rate
		-	+			
<40y	-	54	13	67	0.04	69.70%
	+	27	38	65		
	Subtotal	81	51	132		
≥40y	-	50	9	59	0.82	79.59%
	+	11	28	39		
	Subtotal	61	37	98		
Underweight	-	6	3	9	_	62.50%
	+	3	4	7		
	Subtotal	9	7	16		
Normal BMI	-	75	15	90	0.49	74.57%
	+	29	54	83		
	Subtotal	104	69	173		
Overweight /Obese	-	23	4	27	0.75	75.61%
	+	6	8	14		
	Subtotal	29	12	41		
All	-	104	22	126	0.05	73.91%
	+	38	66	104		
	Subtotal	142	88	230		

-: Follow-up (BI-RADS = 1/2); +: Recall (BI-RADS = 3/4); Underweight: BMI < 18.5 kg/m^2^; Normal: 18.5 ≤ BMI ≤ 23.9 kg/m^2^; Overweight/Obese, BMI > 23.9 kg/m^2^. AIBUS, Artificial Intelligent Breast Ultrasound; HHUS, hand-held ultrasound; BMI, body mass index.

### 3.3 Lesion detection and characterization between AIBUS with HHUS

In both examinations where breast information was completely recorded, 440 participants had lesions. The thickness of the breast measured by AIBUS (9.42 ± 3.36mm) was 0.73 (95% *CI* (0.36, 1.10), *P* < 0.001) mm thinner than that by HHUS (10.15 ± 3.88mm) according to the results of paired t-test. The intraclass correlation coefficient was 0.575 (95% *CI* (0.487, 0.647), *P* < 0.001), showing a moderate correlation. 236 lesions detected by both devices were matched and AIBUS measured shorter in either long diameter (*P* < 0.001) or short diameter (*P* = 0.001) and deeper from the skin (*P* < 0.001) than HHUS (**Table 3**).

**Table 3:** Comparison of lesion features between HHUS and AIBUS.

Parameters	HHUS			AIBUS			*P*
	Mean ± Sd	M [*P*_25_, *P*_75_]	Range	Mean ± Sd	M [*P*_25_, *P*_75_]	Range	
Long Diameter (mm)	9.58 ± 4.46	8.00 [7.00, 12.00]	(4.00, 29.00)	8.15 ± 3.41	7.35 [5.68, 10.10]	(3.50, 24.90)	<0.001^a^
Short Diameter (mm)	4.76 ± 1.32	4.00 [4.00, 6.00]	(3.00, 7.00)	4.34 ± 1.75	4.00 [3.00, 5.40]	(1.40, 11.40)	0.001^a^
Depth from the skin (cm)	2.03 ± 2.55	0.95 [0.70, 1.50]	(0.11, 13.00)	2.32 ± 2.61	1.10 [0.90, 1.76]	(0.10, 12.00)	<0.001^b^

^a^ Paired t-test, ^b^ Wilcoxon test. AIBUS, Artificial Intelligent Breast Ultrasound; HHUS, hand-held ultrasound.

In the overweight and over 40-year-old subgroups, McNemar’s test showed no difference between the breast density categories by the two examinations (**Table 4**). The agreement reached the highest in the overweight subgroup, with *κ* of 0.73 (95% *CI* 0.66, 0.8), and a consistency rate of 86.36%. The *κ* in the subgroup of underweight was 0.62 (95% *CI* 0.48, 0.76), showing a substantial agreement. The agreements were moderate (0.43, 95% *CI* (0.38, 0.48)) in the two subgroups under 40 years and with normal BMI, both having the lowest consistency rates of 71.1% (**Figure 3**).

**Table 4:** Comparison of breast density category between HHUS and AIBUS.

Subgroups	HHUS	AIBUS		Subtotal	*P*	Consistency rate
		-	+			
<40y	-	67	11	78	<0.001	71.14%
	+	60	108	168		
	Subtotal	127	119	246		
≥40y	-	78	14	92	0.081	79.38%
	+	26	76	102		
	Subtotal	104	90	194		
Underweight	-	9	0	9	0.063	80.77%
	+	5	12	17		
	Subtotal	14	12	26		
Normal BMI	-	97	21	118	<0.001	71.17%
	+	73	135	208		
	Subtotal	170	156	326		
Overweight /Obese	-	39	4	43	0.388	86.36%
	+	8	37	45		
	Subtotal	47	41	88		
All	-	145	25	170	<0.001	74.77%
	+	86	184	270		
	Subtotal	231	209	440		

- : non-dense; +: dense; Underweight: BMI < 18.5 kg/m^2^; Normal: 18.5 ≤ BMI ≤ 23.9 kg/m^2^; Overweight/Obese: BMI > 23.9 kg/m^2^. AIBUS, Artificial Intelligent Breast Ultrasound; HHUS, hand-held ultrasound; BMI, body mass index.

The average total time of HHUS was 10.63 ± 4.3 min, range (2.2, 18.3) min, whereas the average total time of AIBUS was 9.59 ± 3.05min, range (5.23, 19.57) min. The results of the t-test revealed that the average time consumption of the two inspections was statistically different. AIBUS saved an average of 1.03 (95% *CI* (0.57, 1.50)) minutes per case (*P* < 0.001).

## 4 Discussion

HHUS, as a front-line screening tool in the detection and characterization of breast lesions (15), is mainly limited by high dependence on radiologists and irreproducible images (16, 17). As a result, some innovative imaging technologies have reached the market as critical alternative tools (18). However, innovative imaging technology often lacks evaluation and substantiation, and its suitable population and medical use scenarios remain unclear. This study is the first to compare AIBUS and HHUS in a healthy population.

An overall satisfaction level of 70% was attained with the AIBUS image quality. Only one-third of AIBUS images met the high-quality standard set out in this study when we neglected to analyze the HHUS image quality. However, in actual use, it is doubtful that HHUS will always be successful. Therefore, the disparity between the two ultrasound images is overestimated. Under a programmed and complete scanning path of the robotic arm, frame loss may be explained by the discontinuous contact of the ultrasonic probe due to respiratory motion, which can be avoided when subjects breathe calmly. We could modify the pressure transducer on the robotic arm to apply uniform and constant compression to the breast to eliminate the artifactual posterior shadowing behind the glandular layer. Identifying these defects associated with breast ultrasonography and addressing these limitations contributes to obtaining high-quality images, which can improve the diagnostic potential of AIBUS.

We found a moderate agreement in the classification of the “recall/follow-up” (*κ* = 0.47) category in good-quality images, with no significant differences between AIBUS and HHUS. Given its positioning in the management of asymptomatic women in screening practice, it is worth noting that the final assessment category should be considered as a critical indicator for evaluating such products. The results of subgroup analysis suggested a better classification of the BI-RADS final recall assessment (*κ* = 0.57) and density category (*κ* = 0.59) when applied to women over 40 years old, indicating a potentially more suitable population under such examination. Similar products described in the literature have been reported to have a kappa value of up to 0.63-0.70 when applied to outpatients who were already pre-diagnosed breast lesions (19, 20). Since a large number of women without breast lesions are also included in the actual screening group, our data may be more persuasive in the application of primary screening for asymptomatic women. We were able to demonstrate the lesion detection capability of AIBUS is comparable to that of a conventional device, in which case thorough documentation of the lesions for re-reading could be considered an advantage of AIBUS. Accurate lesion characterization is critical to the successful use of automated ultrasound. Compared with HHUS, we found that the lesions under AIBUS were shorter in diameter and deeper from the skin. The greater pressure of the AIBUS probe may account for the differences,which were all within 3millimeterss and have no significant impact on clinical conclusions. Similar results and inferences were also found in the comparison of breast thickness.

Given its lower operator dependency, AIBUS showed the advantage of a shorter time to perform the exam and less cost of training specialized radiologists. Without compromising image quality, automatic acquisition equipment can be employed in primary medical institutions to conserve resources. The complete image documentation can be read by radiologists in tertiary institutions, facilitating cross-regional diagnosis. In addition to the high reliance on experience, an excessively short scan time by HHUS may result in poor image quality, which can be avoided by standardized image acquisition.

Maryellen found that combining mammography with AIBUS, compared with mammography alone, significantly improved readers’ detection of breast cancers in women with dense breast tissue without substantially affecting specificity (21). Fernanda compared BI-RADS of HHUS and AIBUS in patients and found the overall concordance was 80.9% (22). Whereas these studies were conducted on patients, our study, which served as a foundation for the use of AIBUS in future population screening, was based on asymptomatic women. Sung et al. summarized the unique display mode, imaging features, and artifacts in ABUS. The coronal view is the unique display mode of ABUS, which shows the entire breast anatomy (23). The display mode of AIBUS used in was a transverse section like HHUS. In addition, compared to its counterpart (22), which needs the operator to manually place the scanning probe, AIBUS requires less operator effort and it scans automatically with the press of a single key. This reduces labor expenses for operators even further and facilitates the promotion and use of AIBUS in basic medical institutions that provide screening services. As a new technology, AIBUS should be utilized responsibly with a thorough grasp of its advantages and limitations. Our results provided evidence to quantify the application value of the prospective screening tool and would help improve breast cancer screening decisions.

### 4.1 Limitations

First, only images of good-quality AIBUS were included in the comparison of recall/follow-up classification. It is suggested in practical screening management, quality control should be realized from a variety of angles. Second, results were not contrasted amongst different radiologists, therefore, interobserver variability was not determined. Furthermore, the lack of follow-up results as a gold standard presented a challenge for this cross-sectional study based on healthy populations. However, the selection bias was avoided. Whether it is suitable for various screening settings requires further study in multicenter populations.

## 5 Conclusion

Moderate agreement was obtained in describing the BI-RADS final recall assessment and breast density category. Acceptable differences were obtained for reporting lesion size and location. Such innovative products with auxiliary diagnostic functions should have post-market evaluation when used among the screening population.

## Data availability statement

The raw data supporting the conclusions of this article will be made available by the authors, without undue reservation.

## Ethics statement

The studies involving human participants were reviewed and approved by Sichuan University Medical Ethics Committee, Sichuan University. The patients/participants provided their written informed consent to participate in this study.

## Author contributions

BX, WL, and JL designed the study protocol. BX and XC conceived of the analysis. BX wrote the first draft of the manuscript with feedback from all other authors. LT, YJ, MW, and BL were involved in collecting and managing data. All authors contributed to the article and approved the submitted version. 

## Supplementary material

Supplementary Table 1 Evaluation of AIBUS image quality.

Supplementary Table 2 Number of lesions detected by HHUS and AIBUS.
