# Supplementary material for: Evaluation of artificial intelligent breast ultrasound on lesion detection and characterization compared with hand-held ultrasound in asymptomatic women
Source: Front Oncol. 2023 Jun 15;13:1207260. doi: 10.3389/fonc.2023.1207260 (PMC10311017; doi:10.3389/fonc.2023.1207260)
Supplement: Supplementary Table 1 — Evaluation of AIBUS image quality. [file Table_1.docx]

Supplementary Material

Evaluation of Artificial Intelligent Breast Ultrasound on lesion detection and characterization compared with hand-held ultrasound in asymptomatic women

Bin Xu, Jiayuan Li *

*** Correspondence:** Jiayuan Li: [lijiayuan@scu.edu.cn](mailto:lijiayuan@scu.edu.cn)

# Supplementary Figures and Tables

## Table S1 Evaluation of AIBUS image quality

| Dimensions | Mean±Sd | Range | Degree of satisfaction |
| --- | --- | --- | --- |
| Integrity | 3.66±0.92 | (1, 5) | 0.73 |
| Clarity | 3.39±0.72 | (1, 5) | 0.68 |
| Effective image proportion | 3.45±0.87 | (1, 5) | 0.69 |
| Total | 10.50±1.70 | (3,15) | 0.70 |

Degree of satisfaction =Mean score/Total score

‘Integrity’ was the extent to which the reviewer observed the bilateral breast swept in the AIBUS image. A score of 1 was no coverage at all, 2 was mostly no coverage, 3 was fair, 4 was mostly coverage, and 5 was complete coverage.

‘Clarity’ was the subjective assessment by the reviewer of the degree of similarity on the clarity of the AIBUS image compared to the HHUS. A score of 1 was very unclear, 2 was unclear, 3 was fair, 4 was clear, and 5 was very clear.

‘Effective image proportion’ refers to the reviewer’s subjective assessment of the proportion of valid AIBUS images to all total frames. A score of 1 represents less than 20%, a score of 2 represents 20% to 39%, a score of 3 represents 40% to 59%, a score of 4 represents 60% to79%, and a score of 5 represents 80% or more

| Table S2 Number of lesions detected by HHUS and AIBUS | | |
| --- | --- | --- |
| Number of lesions | HHUS (N, (%)) | AIBUS (N, (%)) |
| 0 | 128 (55.7%) | 143 (62.2%) |
| 1~3 | 88 (38.2%) | 78 (33.9%) |
| 4~6 | 12 (5.2%) | 7 (3.0%) |
| 7~9 | 2 (0.8%) | 2 (0.8%) |
| Total | 230 (100%) | 230 (100%) |

AIBUS: Artificial Intelligent Breast Ultrasound; HHUS: hand-held ultrasound.
